# Formation of βTC3 and MIN6 Pseudoislets Changes the Expression Pattern of Gpr40, Gpr55, and Gpr119 Receptors and Improves Lysophosphatidylcholines-Potentiated Glucose-Stimulated Insulin Secretion

**DOI:** 10.3390/cells9092062

**Published:** 2020-09-09

**Authors:** Anna Drzazga, Eliza Cichońska, Maria Koziołkiewicz, Edyta Gendaszewska-Darmach

**Affiliations:** Institute of Molecular and Industrial Biotechnology, Faculty of Biotechnology and Food Sciences, Lodz University of Technology, Stefanowskiego 4/10, 90-924 Lodz, Poland; eliza.cichonska@dokt.p.lodz.pl (E.C.); maria.koziolkiewicz@p.lodz.pl (M.K.)

**Keywords:** lysophosphatidylcholine (LPC), insulin secretion, MIN6, βTC3, pseudoislets, G protein-coupled receptors (GPCRs)

## Abstract

The impaired spatial arrangement and connections between cells creating islets of Langerhans as well as altered expression of G protein-coupled receptors (GPCRs) often lead to dysfunction of insulin-secreting pancreatic β cells and can significantly contribute to the development of diabetes. Differences in glucose-stimulated insulin secretion (GSIS) are noticeable not only in diabetic individuals but also in model pancreatic β cells, e.g., βTC3 and MIN6 β cell lines with impaired and normal insulin secretion, respectively. Now, we compare the ability of GPCR agonists (lysophosphatidylcholines bearing fatty acid chains of different lengths) to potentiate GSIS in βTC3 and MIN6 β cell models, cultured as adherent monolayers and in a form of pseudoislets (PIs) with pancreatic MS1 endothelial cells. Our aim was also to investigate differences in expression of the GPCRs responsive to LPCs in these experimental systems. Aggregation of β cells into islet-like structures greatly enhanced the expression of *Gpr40*, *Gpr55*, and *Gpr119* receptors. In contrast, the co-culture of βTC3 cells with endothelial cells converted the GPCR expression pattern closer to the pattern observed in MIN6 cells. Additionally, the efficiencies of various LPC species in βTC3-MS1 PIs also shifted toward the MIN6 cell model.

## 1. Introduction

Diabetes is one of the most abundantly spread metabolic diseases worldwide and one of the leading causes of death, remaining without effective treatment methods. The antidiabetic monotherapies are still deficient in maintaining long-term glycemic regulation and are coupled with side effects [1]. Therefore, there is a constant need for the development of alternative medication [2]. On the other hand, selecting appropriate cell models for in vitro development of pancreatic islet model which would resemble natural islets of Langerhans more closely, is also challenging. The majority of research completed to date has used rodent pancreatic β cell lines due to their immortality and stimulus-induced insulin-secretion [3]. Murine (e.g., MIN6, NIT-1, βTC, and βHC clones) and rat (e.g., RIN, INS-1) insulinoma cells are most often applied [3,4,5,6,7]. However, several investigated β cell lines present a limited ability to produce insulin (e.g., some subclones of βTC and βHC) regardless of the applied method of cell line generation [8,9,10]. For example, the oncogenic SV40 large T antigen was utilized to generate βTC clones demonstrating little glucose responsiveness [8] as well as MIN6 β cells displaying near-normal glucose-stimulated insulin secretion (GSIS) ratio corresponding to properties of healthy functioning β cells [11]. MIN6 is one of the most commonly used models of pancreatic β cells, as indicated by estimated publication numbers (over 1600 published research results). Nonetheless, the βTC subclones have served as research material in projects published around 140 times so far.

Although culturing β cells as monolayers have been the most frequently used, βTC clones and MIN6 cell lines can spontaneously form three dimensional aggregates (pseudoislets, PIs) that resemble primary islets of Langerhans in size and appearance. The main advantage of PIs is the maintenance of some degree of the cell to cell interactions present in vivo, which allows for improved basal insulin production, as well as GSIS compared to corresponding monolayers [12,13,14,15]. Spelios et al. conducted several projects co-culturing the βTC3 cell model of impaired insulin secretion and islet-derived endothelial cells. Free-floating PIs showed improved insulin production and enhanced glucose responsiveness [7,16].

Pseudoislets have been shown to release an enhanced insulin amount in response to a variety of stimuli, including physical or pharmacological depolarizing agents and nutrients [12,17,18,19]. However, to date, no attempts have been made to exploit the effect of PI formation on G protein-coupled receptors (GPCRs) expression and/or activation, although modulating the activity of GPCRs is an essential approach in modern drug discovery. An estimated 30–40% of FDA-approved drugs target or signal GPCRs [20]. To the best of our knowledge, the only example of GPCRs studied so far in pseudoislet models is the glucagon-like peptide-1 receptor (GLP1R). Green et al. showed that the GLP1R gene was significantly upregulated in pseudoislets formed by human 1.1B4 β cells compared to monolayers [21]. Similarly, higher levels of GLP1R accompanied with better responsiveness to exendin-4 were demonstrated in PIs formed by co-culture of the human EndoC-βH1 β-cell line and murine MS1 endothelial cells [22]. Among pancreatic GPCRs, Free Fatty Acid Receptor 1 (FFAR1, also referred to as GPR40) and GPR119 are popular examples of receptor targets that have received recent attention in the field of diabetes therapeutics [23,24,25,26,27,28,29]. In turn, McKillop et al. were one of the precursors to show that cannabinoid receptor–GPR55 also plays a role in direct modulation of insulin secretion [30,31]. Apart from cannabinoids and lysophosphatidylinositol, lysophosphatidylcholine (LPC) has also been evidenced as a GPR55 agonist [32]. Very recent papers define GPR55 as a new anti-diabetic target [33].

Our group has recently shown that in the MIN6 cell line all three GPCRs (GPR40, GPR55, and GPR119) can be efficiently activated by LPC, which is the most abundant lysophospholipid in human plasma [28]. LPCs appeared as insulin secretagogues also in βTC3 cells [29]. The limitation of natural LPCs is their instability when administered in vivo. We have overcome this barrier with the development of phosphorothioate LPC analogues significantly resistant towards enzymatic degradation due to two incorporated modifications: A methoxy group in *sn*-2 position and a hydrophilic phosphate head modified by a sulfur atom [34]. When natural and synthetic LPCs were used to stimulate insulin production in different cell models (MIN6 and βTC3), it was found that different β cells preferred LPCs bearing fatty acid chains of varying lengths [28,29]. Considering these differences, the work presented hereby attempts to examine the ability of various LPC agonists of GPR40, GPR55, and GPR119 receptors to potentiate GSIS in co-cultures of MIN6 and βTC3 with the MS1 pancreatic endothelial cell line. Several forms of LPC containing lauroyl (12:0), myristoyl (14:0), palmitoyl (16:0), stearoyl (18:0), and oleoyl (18:1) acyl chains were assessed, including phosphorothioate stabilized analogues. We also used the monolayer and PI models to investigate the expression pattern of GPR40, GPR55, and GPR119 receptors in these experimental systems.

## 2. Materials and Methods

### 2.1. Reagents

Dulbecco’s modified Eagle’s medium (DMEM) containing 4.5 g/L glucose (25 mM), DMEM no glucose, fetal bovine serum (FBS), horse serum (HS), and phosphate-buffered saline (PBS pH 7.4) were obtained from Life Technologies (Carlsbad, CA, USA). Antibiotics (penicillin, amphotericin B, and neomycin), sodium pyruvate, β-mercaptoethanol, d-glucose, HEPES, NaCl, KCl, MgCl_2_:6H_2_O, CaCl_2_:2H_2_O, DMSO, MeOH, HCl, and Bradford reagent were obtained from Merck KGaA (Darmstadt, Germany). Glipizide was purchased from Cayman Chemical (Michigan, MI, USA). A 10 mM stock solution was prepared in DMSO and further diluted in cell culture media. Native LPCs (12:0, 14:0, 16:0, 18:0, and 18:1, respectively depicted as L, M, P, S, and O) were purchased from Avanti Polar Lipids (Alabaster, AL, USA). The 2-O*Me*-phosphorothioate analogues of LPCs tested during the experiments were originally synthesized at the Institute of Molecular and Industrial Biotechnology, Lodz University of Technology as described previously [34] and are further depicted as LT, MT, PT, ST, and OT, with respect to the acyl chain and analogically to their native counterparts. Solid compounds were solubilized in MeOH in 5 mM concentrations further diluted in PBS or cell culture media. Specific antagonists of GPR40 (DC260126, depicted as DC) and GPR55 (CID16020046, depicted as CID) were obtained from Tocris Bioscience (Ellisville, MS, USA). The GPR119 antagonist (depicted as C8) was kindly provided by Pfizer (Groton, CT, USA) [35]. All antagonists were prepared as 10 mM stock solutions in DMSO and applied for cell culture studies at 2 μM working concentrations, as previously [25]. Reagent kits for RNA isolation (GeneMatrix Universal RNA Purification Kit), DNAse, and quantitative RT-PCR (NG dART RT kit) were obtained from EURx (Gdansk, Poland). Primers were obtained Genomed (Warsaw, Poland).

### 2.2. Cell Culture Conditions

Regular cell cultures were conducted in cell line-specified growth media supplemented with antibiotics (1% penicillin/amphotericin B/neomycin), at 37 °C atmosphere of 5% CO_2_.

#### 2.2.1. βTC3 Cell Line

The murine insulinoma βTC3 cells were obtained from the Leibniz Institute DSMZ–German Collection of Microorganisms and Cell Cultures (Braunschweig, Germany). They were maintained in DMEM containing 25 mM glucose, 7.5% HS, and 1.5% FBS.

#### 2.2.2. MIN6 Cell Line

The murine insulinoma MIN6 cells were kindly provided by Prof. Peter Bergsten (Uppsala University, Uppsala, Sweden) by permission of Dr. Jun-ichi Miyazaki (Division of Stem Cell Regulation Research, Osaka University, Osaka, Japan). The culture medium consisted of DMEM containing 25 mM glucose, 10% fetal bovine serum, 50 μM β-mercaptoethanol.

#### 2.2.3. MS1 Cell Line

MS1 cells were obtained from American Type Culture Collection ATCC (Rockville, MD, USA). They were cultured in DMEM containing 25 mM glucose and 5% FBS.

### 2.3. Pseudoislets Formation

For PI formation, co-cultures were prepared by seeding 2 × 10^5^ of β cells/well (either βTC3 or MIN6 cell line) and 6 × 10^5^ cells/well MS1 pancreatic endothelial cell line in a 6-well tissue culture plate, as suggested by Spelios et al. [16]. Additionally, PIs containing only one cell line (βTC3, MIN6, or MS1) were prepared analogously, by seeding 8 × 10^5^ cells/well of a 6-well plate. The co- and monocultured PIs in DMEM containing 25 mM glucose, 10% FBS, and 1 mM sodium pyruvate for 6-days before the experiments, however, they can be maintained for at least 14-days. The formation of PIs was monitored under a Nikon Eclipse TS 100 microscope with the NIS-Elements AR 3.22.14 visualization software.

### 2.4. Stimulation of Ins1 Expression in Adherent βTC3 and βTC3-MS1 Pseudoislets

Before the experiment adherent βTC3 and βTC3-MS1 pseudoislets were cultured for 24 h in DMEM with 1 mM glucose and 0.5% FBS. The next day, the βTC3 monolayer and the PIs were supplemented with glucose to obtain a 25 mM concentration, and/or with 50 μM Glipizide. The PIs were incubated for 2 h before RNA isolation.

### 2.5. Glucose-Stimulated Insulin Secretion (GSIS)

In the case of GSIS experiments, cultures of β cell lines, either in adherent monoculture or in the form of floating PIs, were incubated at 37 °C in a calcium buffer (25 mM HEPES, 125 mM NaCl, 6 mM KCl, 1.2 mM MgCl_2_:6H_2_O, 1.3 mM CaCl_2_:2H_2_O, pH 7.4) supplemented with 2 mM (low) glucose for 60 min. After the initial preincubation, the buffer portion was changed and supplemented with investigated compounds (native or modified LPCs or Glipizide) used at 10 μM concentration with or without receptor antagonists (applied at 2 μM concentration), and the cells were incubated at low glucose conditions for 30 min. Subsequently, the buffer supernatants were collected, and the cells were further incubated in the calcium buffer containing respective investigated compounds at 10 μM concentration, at 20 mM (high) glucose conditions, for another 30 min. The buffer samples were collected again and, together with the samples from low glucose conditions, were subjected to measurement of the amount of secreted insulin via competitive ELISA [36]. The cells were trypsinized, resuspended in a glucose-free calcium buffer, and lysed via repetitive freeze-thawing. The obtained cell lysates were centrifuged to remove the cell debris and used to assess the amount of total protein by the Bradford method [37]. In the case of Glipizide stimulation, the cell lysates were also used to measure the intracellular insulin content via competitive ELISA [36]. The amounts of secreted and intracellular insulin were normalized to the total protein content in the respective cell lysates. In the case of adherent cells, the experiments were conducted in 24-well plates, whereas the PIs (500–1000) were placed in Eppendorf tubes and subsequent changes of buffer portions via centrifugation at 300*× g* for 45 s.

### 2.6. RNA Isolation and Quantitative Reverse Transcription PCR Analysis

Total RNA was extracted and purified according to the GeneMatrix Universal RNA Purification Kit protocol. The quality of isolated RNA was verified with electrophoresis and absorbance reads (260, 280, and 230 nm). Portions of 0.5 μg total RNA were used in RT-qPCR according to the NG dART RT protocol (including treatment with DNAse), in CFX96 Touch Real-Time PCR Detection System (BioRad, California, CA, USA). Primers (Table 1) were designed using the NCBI’s Primer-BLAST software (National Center for Biotechnology Information) based on receptors’ sequences from the GenBank database. β-actin (*Actβ*) gene was chosen as the house-keeping control. Samples reaching the threshold cycle for Actβ at the level of 19.0 ± 0.5 were subjected to further analysis. The qPCR products were identified based on their melting temperature calculated in the OligoCalc online software and electrophoresis. The purity of cDNA samples, as well as lack of any off-targets for the chosen primers, were additionally confirmed by running qPCR in samples without the reverse transcriptase treatment.

### 2.7. Statistical Analysis

Results are presented as means of 3–4 repeated experiments (3–8 biological repeats each) ± standard error of the mean (SEM). Groups of data were compared using one-way ANOVA with the Bonferroni post-hoc test. Statistical significance of the obtained results was determined with GraphPad Prism version 8.4 (GraphPad Software, La Jolla, CA, USA).

## 3. Results

### 3.1. Comparison of the Responsiveness of βTC3 and MIN6 Monolayers as Well as Pseudoislets to Glipizide

**Figure 1:**
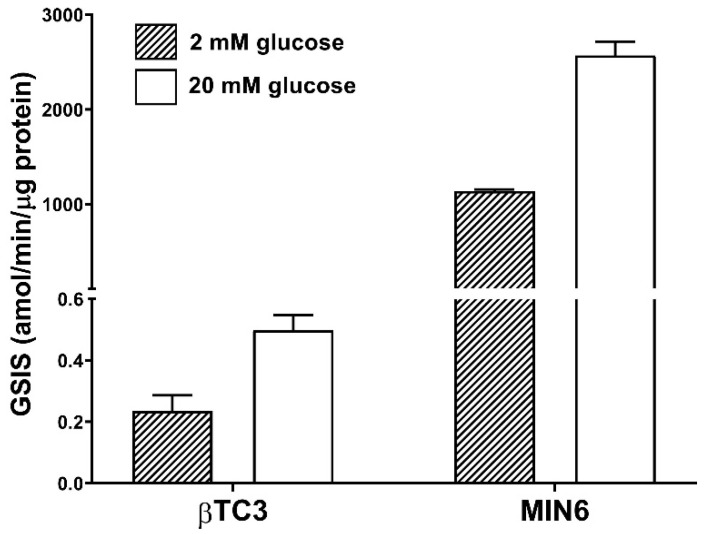
The efficiency of glucose-stimulated insulin secretion (GSIS) in βTC3 and MIN6 adherent cultures.

We have chosen MIN6 and βTC3 cell lines due to their different glucose-responsive insulin secretion. Initial GSIS experiments confirmed βTC3 to be a much less efficient producer of insulin compared to MIN6. Absolute amounts of secreted insulin normalized per mass and per time unit in the case of MIN6 were ca. 5000-fold higher than in the case of βTC3 (Figure 1). Both cell lines present similar responsiveness to glucose stimulation secreting 2–3-fold more insulin at high glucose conditions (20 mM) compared to low glucose conditions (2 mM).

**Figure 2:**
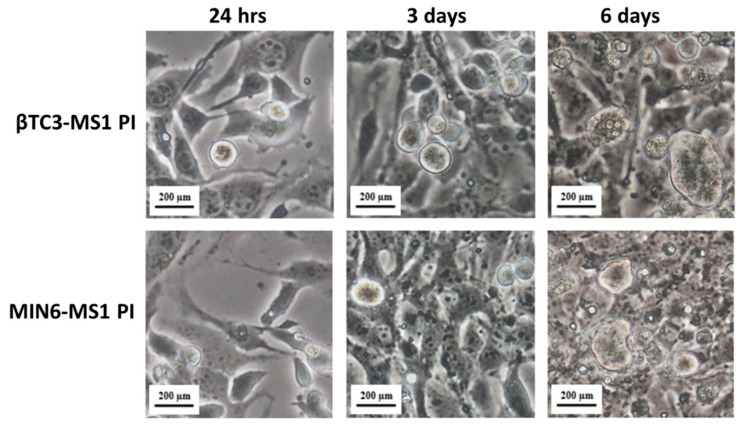
Microscopic view (400-fold magnification) of pseudoislets (PIs) formed from adherent βTC3 or MIN6 insulinoma cells mixed with MS1 pancreatic endothelial cells, after 24 h, three and six days of co-culture.

β cells require homotypic cell-cell interaction to function correctly in vitro. Therefore, most studies use pure populations of insulin-secreting cells to generate functional pseudoislets [13,38]. It was shown that homotypic interactions within MIN6 PIs in which cells are assembled in a three-dimensional configuration were sufficient to significantly enhance secretory responsiveness to nutrients and non-nutrient stimuli when compared to equivalent cells configured as monolayers [12]. In vivo, however, pancreatic islets are perfused by a dense, specialized microvasculature with endothelial cells governing several cellular and pathophysiological processes associated with the pancreatic tissue functional PIs [39]. Since the formation of pseudoislet phenotype allows for the combination of more than one type of cells, Spelios et al. developed a co-culture system for the engineering of murine PIs using βTC3 cells and MS1 endothelial cells [16]. MS1 is a pancreatic islet microvascular endothelial cell line established from mouse C57BL/6 strain and transduced with the SV40 large T antigen. The line retains many properties of endothelial cells including uptake of acetylated LDL and expression of both Factor VIII related antigen and VEGF receptor [40]. Endothelial cells-induced βTC3 PIs showed improved glucose-stimulated insulin secretion and *de novo* deposition of critical extracellular matrix proteins around the PIs [16]. As a consequence, we applied βTC3 and MIN6 cells co-culture with the MS1 cell line (1:3 ratio) to facilitate PI formation. In both cases, the formation of free-floating roundish shape PIs with a diameter of up to ca. 200 μm was observed six days after seeding (Figure 2). These parameters correspond to the sizes of pseudoislets obtained by other research groups [14,16,41]. The functionality of MIN6 cells in the 3D co-culture with endothelial cells is presented hereby for the first time.

**Figure 3:**
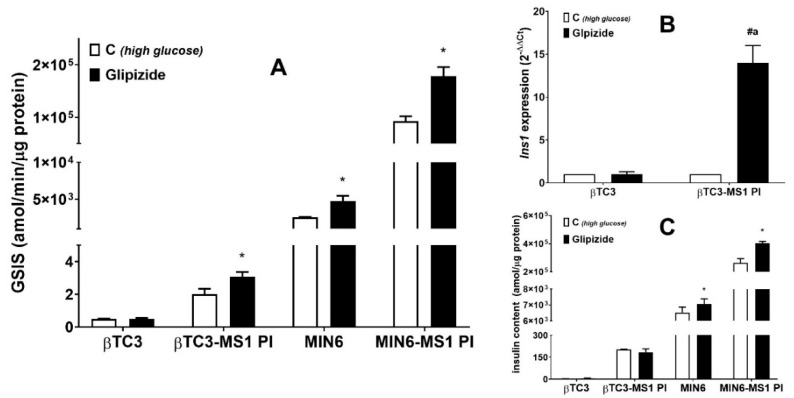
Responsiveness of βTC3 and MIN6 cell lines while maintained in the form of adherent monocultures (βTC3 and MIN6, respectively) or three-dimensional (3D) pseudoislets (PI) in co-cultures with MS1 cells (βTC3-MS1 PI and MIN6-MS1 PI, respectively) to Glipizide. (**A**) GSIS in adherent cells or PIs treated with Glipizide. (**B**) Expression of *Ins1* in βTC3 and βTC3-MS1 PI after treatment with Glipizide. (**C**) Insulin content in adherent cultures and MS1-supplemented PIs of βTC3 and MIN6 treated with Glipizide. For the *p*-value less than 0.05 results are regarded as significantly different from the corresponding control (high glucose) conditions (**C**) * or from adherent cultures treated with Glipizide ^a^.

The key measure of β cell functionality is the stimulus-induced GSIS. We employed this method to monitor insulin secretion from βTC3 and MIN6 monolayer cultures and the corresponding PIs. Initially, the responses to known stimuli, Glipizide, was tested. Glipizide is a second-generation anti-diabetic sulfonylurea drug partially closing ATP-sensitive K^+^ channels in the β cell plasma membrane. This blockade results in depolarization of cells, which opens the voltage-gated calcium channels, and subsequently leads to insulin release [42]. Glipizide should stimulate insulin secretion above the control level in healthy-functioning β cells [43]. MIN6-MS1 PIs, as well as MIN6 monolayer cultures, responded with enhanced insulin production at high glucose conditions as compared to the control (ca. 2-fold) when treated with 10 μM Glipizide. βTC3 monocultures failed to respond to Glipizide while βTC3-MS1 PIs presented ca. 1.5-fold increase (Figure 3A). Therefore, we show for the first time that the co-culture of βTC3 with MS1 cells recovers Glipizide potentiation of GSIS. Subsequently, we sought to determine whether MS1-induced βTC3 PIs express insulin at levels comparable to monolayer cells. Quantitative RT-PCR studies showed that βTC3-MS1 PIs preincubated with Glipizide showed a highly increased expression of *Ins*1 mRNA, with no change in βTC3 monolayer (Figure 3B). However, exposure of βTC3 monolayer and βTC3-MS1 PIs to Glipizide did not affect the insulin content (Figure 3C), which suggests the drug stimulates the insulin release without significant alteration to its intracellular amount. A similar observation was made in the case of adherent MIN6 where exposure to Glipizide resulted in a minor 10% increase in insulin content compared to the untreated control cells. Only in the case of MIN6-MS1 PIs Glipizide caused a 40% increase in insulin content.

Importantly, the sole formation of PIs improved both βTC3 and MIN6 β cell function resulting in higher glucose responsiveness compared to adherent monocultures (4-fold and 36-fold elevated insulin secretion in high glucose conditions, respectively, Figure 3A). Our observation is consistent with the results demonstrated by Spelios et al. (increased baseline insulin secretion in response to glucose stimulation in the case of βTC3-MS1 PIs [16]).

### 3.2. Comparison of Insulin Secretory Activity of βTC3 and MIN6 Monolayers in Response to LPC with Various Acyl Chain Lengths

**Figure 4:**
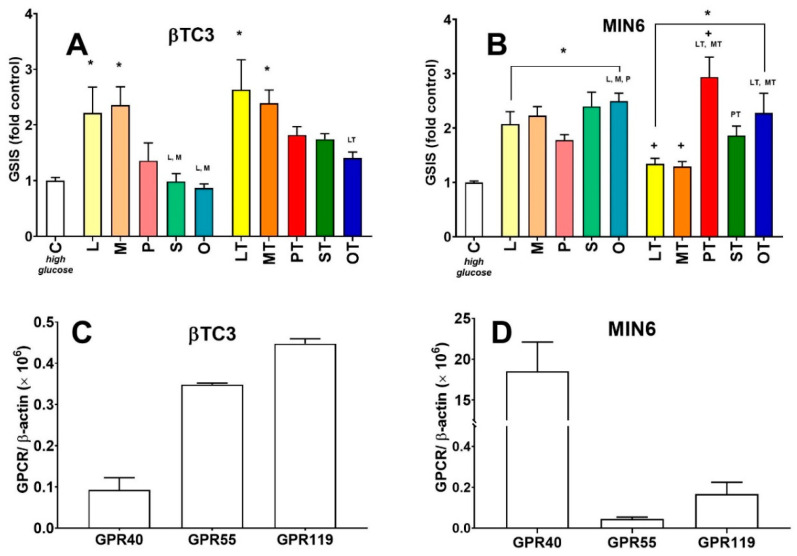
The potentiatory effect on GSIS of native lysophosphatidylcholines (LPCs) (L, M, P, S, O) and their phosphorothioate analogues (LT, MT, PT, ST, OT) in βTC3 (**A**) and MIN6 (**B**) in comparison to GPR40, GPR55, and GPR119 expression patterns in these cell lines (**C**: In βTC3; **D**: In MIN6). GSIS experiments stimulated with native and modified LPCs are presented for high glucose conditions. For the *p*-value less than 0.05 results are regarded as significantly different from control (high glucose) conditions (**C**) * or from the corresponding native LPC ^+^. Statistically significant difference was also assessed between compounds bearing acyl chains of different lengths (either within native or synthetic compound groups), and *p* < 0.05 was depicted by a compound abbreviation.

Next, we decided to examine the GSIS responsiveness of βTC3 and MIN6 monolayer cultures to GPCR agonists acting with different mechanisms of action than Glipizide. We and others have shown that lysophosphatidylcholines facilitate GSIS recognizing GPR119, GPR40, and GPR55 receptors highly expressed on β cells of pancreatic islets [6,28,29,32]. We show that apart from insulin secretory efficiency both pancreatic β cell lines studied hereby respond differently to stimulation with unmodified and modified LPCs. The set of investigated LPCs involved natural LPC 12:0 (L), LPC 14:0 (M), LPC 16:0 (P), LPC 18:0 (S), and LPC 18:1 (O) and corresponding phosphorothioate LPC analogues (LT, MT, PT, ST, OT, respectively). βTC3 secreted the greatest amounts of insulin at high glucose conditions (ca. 3-fold of the control level) when stimulated with LPC molecules bearing medium-chain fatty acids (12:0-L and LT; 14:0-M and MT). For the remaining compounds, there was no statistically significant difference observed with respect to the control conditions. However, the general tendency shows that the longer the fatty acyl, the lesser the GSIS stimulatory effect in βTC3 (Figure 4A). In MIN6 cells, the entire set of native and modified compounds significantly potentiated the level of GSIS above the control level (from 1.5- to 3.5-fold, Figure 4B). In this case, LPCs bearing longer acyl chains tended to stimulate MIN6 cells stronger than those bearing medium acyl chains. PT was the strongest potentiator of the entire set (ca. 3-fold control) as published previously [28] whereas S, O, and OT present similar efficiency as GSIS potentiators in MIN6.

In an attempt to identify possible mechanisms through LPCs that could mediate their activity in both cell lines, mRNAs encoding LPC-responsive GPCRs were quantified. Indeed, differences at the level of GPCR expression between the cell lines were observed. βTC3 presents similar amounts of *Gpr55* and *Gpr119*, whereas *Gpr40* was about 5-fold less abundant (Figure 4C). On the contrary, in MIN6 cells, *Gpr40* was highly expressed, reaching a 200-fold abundance of *Gpr55* and 100-fold abundance of *Gpr119* (Figure 4D). Additionally, when mRNA levels were expressed as a ratio with β-actin, *Gpr40* was at least 200-fold enriched in MIN6 compared to βTC3 cells.

**Figure 5:**
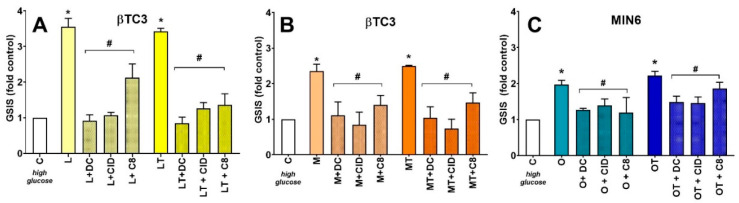
The G protein-coupled receptor (GPCR)-specific activity of most efficient GSIS potentiators in βTC3 (**A**,**B**) and MIN6 (**C**). GSIS experiments stimulated with native and modified LPCs are presented as fold control for 20 mM glucose conditions. DC, CID, and C8 correspond to antagonists of GPR40, GPR55, and GPR119, respectively. For the *p*-value less than 0.05 results are regarded as significantly different from control (high glucose) conditions (**C**) * or from the corresponding compound treatment after application of receptor antagonists ^#^.

The strongest potentiators of insulin secretion in both cell lines (in the case of βTC3 cells L, LT, M, MT whereas in the case of MIN6 O and OT) were tested for their activity related to interaction with the receptors mentioned above involved in insulin secretion (Figure 5). PT being the most potent GSIS inducer in MIN6 cells was not studied hereby since our previous studies revealed that this LPC molecule interacted with GPR40, GPR55, and GPR119 in terms of insulin secretion. In the present study, we demonstrated that the effects of all the applied compounds, as in the case of PT, were mediated by activation of all the three receptors irrespectively of the cell line, type of acyl chain, and/or the presence of phosphorothioate modification. We observed that the addition of DC260126 (a selective antagonist of GPR40), CID16020046 (a selective antagonist of GPR55), and C8 (a selective antagonist of GPR119) blocked GSIS potentiation evoked by LPCs.

### 3.3. Comparison of Insulin Secretory Activity of βTC3 and MIN6 Pseudoislets in Response to LPC with Various Acyl Chain Lengths

**Figure 6:**
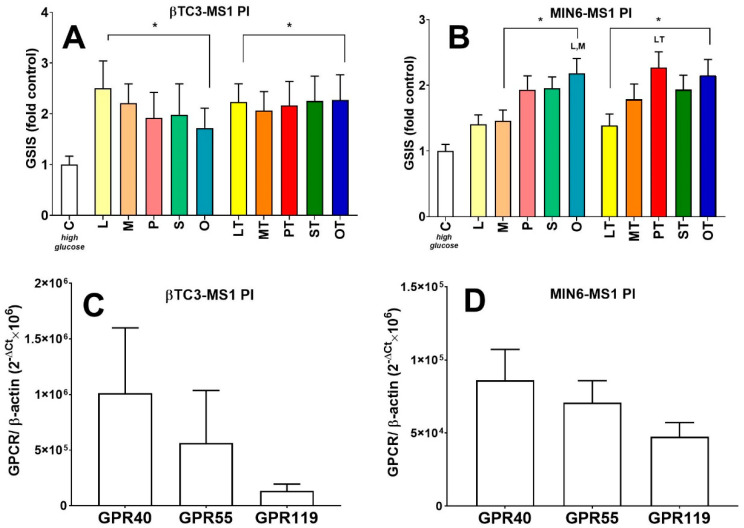
The potentiatory effect on GSIS of native LPCs (L, M, P, S, O) and their phosphorothioate analogues (LT, MT, PT, ST, OT) in βTC3-MS1 (**A**) and MIN6-MS1 PIs (**B**) in comparison to GPR40, GPR55, and GPR119 expression patterns in these PIs (**C**: In βTC3-MS1; **D**: In MIN6-MS1). GSIS experiments stimulated with native and modified LPCs are presented for 20 mM glucose conditions. For the p-value less than 0.05 results are regarded as significantly different from control (high glucose) conditions (**C**) * or from the corresponding native LPC ^+^. Statistically significant difference was also assessed between compounds bearing acyl chains of different lengths (either within native or synthetic compound groups), and *p* < 0.05 was depicted by a compound abbreviation.

In a subsequent series of experiments, insulin responses to unmodified and modified LPCs were studied in βTC3 and MIN6 pseudoislets formed as a co-culture with pancreatic endothelial cells. βTC3-MS1 PIs responded with a statistically significant increase in insulin secretion when stimulated with the entire set of LPCs. It may be noticed that natural LPCs bearing shorter fatty acyls were stronger GSIS potentiators, as in the case of the βTC3 monoculture, although this was not statistically significant. The most striking difference between βTC3 monoculture and βTC3-MS1 was seen for long-chain LPCs (P, S, O) that were able to induce GSIS from pseudoislets (Figure 6A). In contrast, monolayer cells showed no secretory response to P, S, and O LPCs. Phosphorothioate analogues of LPCs seemed to have similar stimulatory efficiency independently of the acyl chain length (Figure 6A) what is also a contrast to βTC3 monoculture. The stimulation of the MIN6-MS1 PIs with LPCs resulted in a statistically significant increase in insulin secretion in the case of all compounds. Still, in the case of native and modified 14:0 LPCs, the increase was not significant statistically. The general tendency of secretion increasing with the length of the acyl chain of the LPC was present for both native and modified LPCs. As in the case of MIN6 monolayer culture, PT elicited the most robust secretory response from pseudoislets (Figure 6B). Overall, the results revealed that the secretory pattern in response to LPC with various fatty acyl chains was comparable for MIN6 and MIN6-MS1 PIs. On the other hand, the reconfiguration of βTC3 monolayers by MS1 endothelial cells into PIs changed the response substantially.

βTC3-MS1 and MIN6-MS1 PIs were also checked for their GPCR expression patterns in terms of GPR40, GPR55, and GPR119 (Figure 6C,D). We showed that aggregation of murine β cells into islet-like structures greatly enhanced the expression levels of all receptors studied. We also observed a striking difference in *Gpr40, Gpr55,* and *Gpr119* mRNA expression patterns between βTC3 and βTC3-MS1 PI models (Figure 4C and Figure 6C). Following pseudoislet formation by βTC3, the profile of three GPCRs studied hereby resembled that of MIN6 and MIN6-MS1 PIs (Figure 4D and Figure 6D) with the highest level of *Gpr40* transcript.

**Figure 7:**
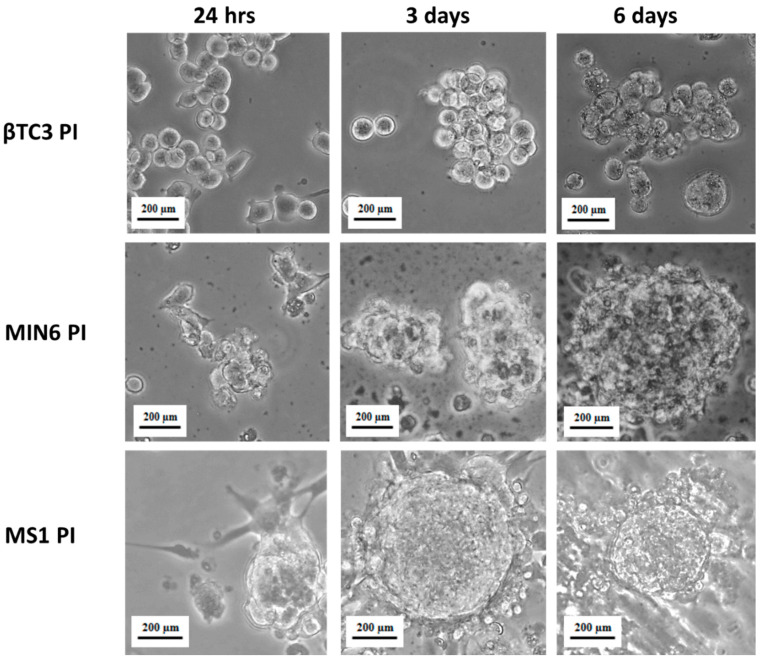
Microscopic view (400-fold magnification) of homotypic pseudoislets formed from βTC3 or MIN6 insulinoma cells or MS1 pancreatic endothelial cells after 24 h, three and six days of co-culture.

It should be noticed that PIs studied hereby were prepared by seeding 2 × 10^5^ of β cells/well (either βTC3 or MIN6 cell line) and 6 × 10^5^ cells/well of MS1 cells. Therefore, the MS1 component of the PIs was also checked for the expression of GPCRs. We evaluated MS1 monolayer cultures as well as MS1 cells maintained in culture as homotypic three-dimensional aggregates. MS1 clusters had a mean diameter of ca. 500 µm after six days of culture, however, they were not floating and started to overgrow with the MS1 monolayer (Figure 7). This observation suggests that the endothelial MS1 cell line needs to be accompanied by another cell type to be able to form a free-floating three-dimensional culture.

**Figure 8:**
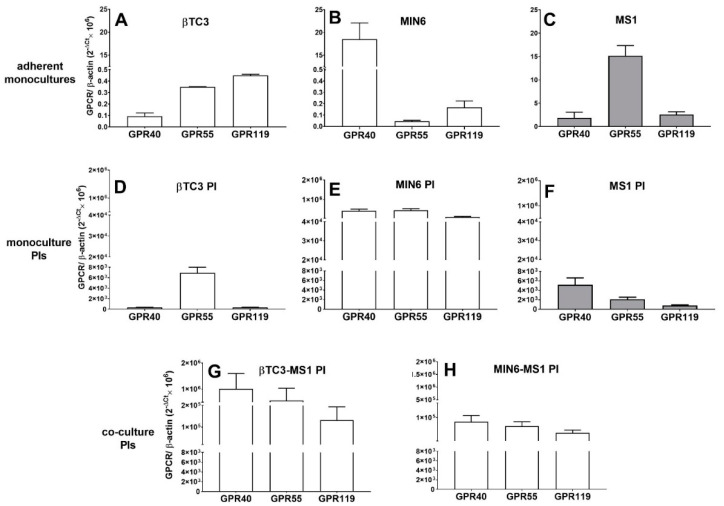
Comparison of GPCR expression patterns in adherent monocultures of βTC3 (**A**), MIN6 (**B**), and MS-1 (**C**) with their respective monocultures in the form of 3D pseudoislets (**D**–**F**, respectively) and the co-cultured pseudoislets: βTC3-MS1 PI (**G**) and MIN6-MS1 PI (**H**).

The mRNA levels of *Gpr55* was significantly higher in the monolayer MS1 cells (Figure 8C) than in monolayer cultures of βTC3 and MIN6. The lowest expression was observed for *Gpr40* mRNA. Since the arrangement of MS1 cells into the 3D architecture could change the reciprocal interactions between endothelial cells [16], we examined the mRNA levels of all three receptors as well. Indeed, we detected significantly augmented expression of all transcripts, whereas *Gpr40* mRNA revealed the highest abundance (Figure 8). Our studies also evaluated whether the formation of homotypic PIs composed solely of β cells would be effective in restoring the high *Gpr40* expression level.

This approach failed in the case of βTC3 PI, confirming the impaired nature of this cell line. Instead, we observed a relatively high level of *Gpr55* transcript (Figure 8). As far as homotypic βTC3 PIs morphology is concerned, the cells did not form roundish 3D structures. Instead, during a six-day culture, they formed an irregular rough and unstable clusters of swollen cells up to 600 μM long and 200 μM wide. Homotypic MIN6 PIs presented significantly elevated expression of all receptors of interest to a similar amount (ca. 20,000-fold of *Gpr40* expression in the MIN6 monolayer). After six-days of culture, the formed PIs had a regular roundish shape in size that varied from 200 to 600 μM in diameter.

## 4. Discussion

Native islets of Langerhans seem to be ‘the gold standard’ model for the investigation of β cell physiology and the development of new therapeutics. Unfortunately, fresh islets culture techniques can rarely be used for more than a few days, limiting their usefulness. As a consequence, rodent insulin-secreting cell lines are usually preferred and most widely used [3]. Importantly, pseudoislet cellular configuration may provide an especially good alternative for isolated islets. The remarkable augmentation in insulin secretion compared with correspondent monolayers in response to secretagogues following homotypic PIs formation was observed with MIN6 [12,14,15,44,45], 1.1B4 [21], or EndoC-βH1 [46,47] cell lines. The enhanced cell-cell contact within PIs was connected with increased calcium signaling, increased E-cadherin levels, and connexin 36 regulating cellular communication and islet architecture [12,45,48]. In addition, proteomics analysis revealed eleven highly enriched pathways in PIs, including those controlling glucose metabolism, cell interaction, and translational regulation. Moreover, during protein profiling, the expression level of Pdx1, a homeobox-containing transcription factor that plays a key role in pancreatic development, and Glut2, the major glucose transporter in β cells, was found to be very similar in MIN6 monolayer cultures and MIN6 homotypic PIs [14]. PIs created with heterotypic co-cultures of insulinoma cell lines and accessory cells such as endothelial [7,16,22], stellate [49], neuroblastoma [50], glucagon- and somatostatin secreting [51] as well as GLP-1 releasing [18] or mesenchymal stem cells [52] have also been explored to provide the added benefit of restoring transformed insulinomas to resemble primary islets.

Hereby, we used PIs generated with MS1 endothelial cells. Such heterotypic aggregates were shown to be superior to monolayer cells and homotypic PIs in terms of improved *de novo* deposition of key extracellular matrix components such as laminin and collagen IV. Additionally, higher expression and altered glycosylation patterns of integrin β1 characteristics for native pancreas were also detected [7,16]. Coculturing of MS1 endothelial cells with the EndoC-βH1 human β cell line affected the expression of key genes involved in the transport of glucose, β cell differentiation, glucose sensing, and insulin processing. Compared with monolayer cells, the MS1-EndoC-βH1 PI formation led to a decreased expression of glucagon mRNA, whereas somatostatin mRNA levels were unchanged. A slight increase in *PDX1* and *MAFA*, a key regulator of GSIS, was also detected in PIs. Additionally, mRNA levels of proinsulin processing proteins (such as proprotein convertase subtilisin/Kexin types 1 and 2 or carboxypeptidase E) were similar between PIs and monolayer cultures and the expression of glucose transporter *GLUT1*, *GLUT2*, and *GLUT3,* as well as the *ABCC8* gene, a component of the K-ATP channel, was increased in PIs. Higher levels of GLP1R were also demonstrated in PIs [22].

However, scarce reports on the expression of G protein-coupled receptors involved in insulin release in PIs exist in the literature. Looking for new strategies for the prevention and treatment of diabetes, GPCRs have attracted attention as potential pharmacological targets, as they regulate pancreatic cell physiology, and have accessible druggable sites at the cell surface [20]. GPR40, GPR55, and GPR119 are crucial targets responsible for the regulation of insulin secretion from β cells in a glucose-dependent manner [6]. The GPR40 mRNA expression was shown to be around 17 times greater in the rat pancreatic islets than in the pancreas as a whole [53]. A very similar ratio was observed in human pancreatic islets and adjacent pancreatic tissue [54]. Importantly, chronic hyperglycaemia causes the abrogated expression of GPR40 and the downregulated release of insulin. *Gpr40* mRNA significantly decreased to 36.8% in islets of hyperglycemic *db/db* mice, which correlates with impaired glucose sensing [55]. Almost total abolishment of *Gpr40* expression in all endocrine cells of the pancreas was observed in diabetic Goto-Kakizaki rats compared with Wistar control islets. In addition, the culture of normal islets isolated from Wistar rats depleted Gpr40 protein expression in β cells being associated with almost total suppression of palmitate-stimulated insulin release [56]. *GPR40* mRNA expression was demonstrated to be significantly reduced in human diabetic islets with respect to non-diabetic islets [57]. GPR40 was also found to be expressed in a large variety of pancreatic β cell lines [53,58]. Consistent with a previous report [53], we found its higher level in MIN6 than in βTC3 cells. On the other hand, *GPR119* and *GPR40* mRNA levels were similar in isolated human and murine pancreatic islets [54]. According to Ekberg et al., both these receptors GPR40 and GPR119 can act in synergy [59]. Likewise, we detected that the level of *Gpr119* in MIN6 PI and MIN6-MS1 PI is comparable to that of *Gpr40*. βTC3-MS1 PI expressed the highest level of *Gpr40* transcript. Taking into consideration *GPR55* gene expression in the heterotypic βTC3-MS1 and MIN6-MS1 pseudoislets, our results show for the first time a high level of GPR55 mRNA level comparable to those of GPR40 and GPR119. Previously, Romero-Zerbo et al. reported a high level of the GPR55 mRNA and protein in rat islets and β cells. However, they did not analyze the *GPR40* and *GPR119* gene expression in these tissues and cells [60]. Similarly, Liu et al. have shown that MIN6 cells, mouse, and human islets express GPR55, again without any comparison to levels of GPR40 and GPR119 receptors [61].

To ascertain the mechanisms responsible for significant changes in the PIs GPCR pattern, we decided to analyze the expression of receptors in MS1 endothelial cells. In 3D structures formed only by endothelial cells, we observed augmented expression of all transcripts with *Gpr40* mRNA reaching the highest abundance. However, expression of all GPCRs studied was not as high as in heterotypic βTC3-MS1 and MIN6-MS1 PIs. On the other hand, homotypic PIs formed solely by βTC3 cells, failed to increase *Gpr40*, *Gpr55*, and *Gpr119* to the level comparable to βTC3-MS1 PIs. Unfortunately, at this moment, we cannot answer whether the changes observed in the βTC3-MS1 co-culture result from altered expression in the β cells or the endothelial cells. However, this observation indicates that MS1 endothelial cells are crucial to restore the functionality of βTC3-MS1 PIs. Islet endothelial cells produce many molecules that impinge on the function and survival of β cells. It was shown that proliferating islet endothelial cells could produce substances that stimulate β cell proliferation, such as hepatocyte growth factor (HGF). This secretion could be induced by soluble signals from the islets, such as vascular endothelial growth factor-A and insulin [62]. Other islet endothelial-derived factors that modulate β cell expansion include endothelin-1 [63], connective tissue growth factor [64], or thrombospondin-1 [65]. Spelios et al., who conducted several projects co-culturing β cell models and islet-derived endothelial cells, showed that MS1, but not βTC3 cells, can produce laminin and collagen IV in vitro. Both proteins were found in and around the PIs, suggesting the continuous deposition of extracellular matrix (ECM) proteins during PI formation [16]. Exposure of β cells to various laminin isoforms has been shown to increase insulin gene transcription and insulin release [62]. In turn, integrin-laminin interactions were shown to affect insulin secretion [66]. Whereas, expression of integrin β1 was detected in many β cell types, including βTC3, βTC3-MS1 PI formation leads to the maturation (glycosylation) of integrin β1 similar to that seen in native pancreas and mouse islets [7]. In turn, integrins, composed of noncovalently linked α and β subunits, form clusters upon which cytoplasmic proteins gather to form a link between ECM and the cell cytoskeleton. The integrin affinity for ECM ligand influences its clustering. For instance, laminin 1 induced clustering of integrins and cytoskeletal-associated molecules into thick, short aggregates, while on laminin 5 these elements were clustered into thin, discontinuous-line structures [67]. In light of this data, the studies shown by Waldeck-Weiermair et al. [68] seems to be extremely interesting. They found that in human endothelial cells upon anandamide/O1602 stimulation, GPR55 clusters with αvβ3 and α5β1 integrins, which is a prerequisite to transmitting signaling towards intracellular downstream targets such as phospholipase PLCγ that, in turn, instigates inositol 1,4,5-triphosphate-triggered intracellular Ca^2+^ mobilization from the endoplasmic reticulum. Interestingly, under conditions of inactive (unclustered) integrins, anandamide binds to the cannabinoid 1 receptor (CB1R), resulting in G_i_ protein-mediated activation of spleen tyrosine kinase (Syk) that inhibits phosphoinositide 3-kinase (PI3K) that represents a key signaling protein in the transduction of GPR55-originated signaling. However, once integrins are clustered, Syk does not further inhibit GPR55-triggered signaling. Therefore, the presence of islet endothelial cells in co-culture with β cells supports not only the proper growth of the latter but may also influence GPCR signaling.

MIN6 pseudoislets were shown to possess increased expression of proteins involved in cell-to-cell communication, especially gap junction and tight junction such as E-cadherin and connexins [14,69]. Recent studies indicate an unprecedented role of GPR40 in facilitating tight junction assembly in airways epithelial cells via 5′ AMP-activated protein kinase (AMPK) activation [70]. It was also reported that linoleic acid-activated GPR40 is responsible for increasing the connexin 43 level in the cell membrane of gastric epithelial cells via the Akt-dependent mechanism [71]. On the other hand, GPR40 is indicated as the receptor influencing gene expression of almost one hundred genes. GPR40-overexpressing RIN-40 β cells treated with linoleic acid showed significantly altered expression of 93 genes associated with olfactory transduction, neuroactive ligand-receptor interaction, MAP kinase signaling pathway, cytokine-cytokine receptor interaction, and regulation of the actin cytoskeleton. More than 30% of the genes were associated with signal transduction and cell proliferation [72]. Summing up the abovementioned observations, we hypothesize that GPR40 may possess the function of master receptor initiating not only FFA- and glucose-stimulated insulin secretion but also structural and functional changes in the cell membrane of pseudoislets-forming cells. It should be underlined that GPR40 is activated by a broad range of medium- to long-chain saturated and unsaturated fatty acids of chain lengths of more than 6 carbons. Miyamoto et al. [64] have reported at least 16 free fatty acids activating the receptor. However, the list of GPR40 ligands is still increasing: Among them is a linoleic gut microbial metabolite (HYA or 10-hydroxy-*cis*-12-octadecenoic acid), omega hydroxylated arachidonic acid metabolite (20-HETE or 20-hydroxyeicosatetraenoic acid), and lysophosphatidylcholines [28,73,74].

Another important observation of the present study is the acyl chain dependent activity of native LPC and their phosphorothioate analogues as GPCR ligands influencing glucose-stimulated insulin secretion. We can hypothesize that different patterns of responses in βTC3 cells and βTC3-MS1 PI are connected with the expression levels of *Gpr40*, *Gpr55*, and *Gpr119* since the formation of βTC3-MS1 PI shifts the expression pattern of GPCRs toward a MIN6 cell line model. Such interpretation, however, must be considered in the context of known limitations. Gpr40, Gpr55, and Gpr119 protein levels and localization would give a more appropriate approach to responsiveness in further studies. We also intend to compare the potential differential response of experimental models studied to agonists different from LPCs, namely selective ligands. Such research would enable us to perform a more detailed characterization of the functionality of the pseudoislets to accurately and undoubtedly disclose the mechanisms responsible for the observed differences between monolayer cultures and pseudoislets.

Additionally, it should be underlined that the native and modified LPC used in our studies have in their structure hydrophilic “head” and lipophilic “tail” which can interact with the cell membrane bilayer. Due to different lengths of fatty acid residues, LPC 12:0, LPC 14:0, and LPC 16:0 interact in a slightly different way with the cell membrane and, possibly, with the transmembrane region or even the intracellular face of the receptors. So far, few studies have been focused on the partitioning of lysophospholipids into membranes. The incorporation of LPCs into lipid bilayers is expected to organize with their polar PC-head groups close to the bilayer interface and their hydrocarbon chain buried into the hydrophobic core of the membrane. The longest acyl-chain LPC 16:0 (displaying the smallest acyl-chain mismatch to the model 1-palmitoyl-2-oleoyl-sn-glycerol-3-phosphatidylcholine (POPC)) gave the weakest perturbation per LPC molecule associated with the membrane. In contrast, the LPC 12:0, with the shortest acyl-chain (showing the most considerable acyl-chain mismatch), displayed the most substantial perturbation [75]. However, membranes used in these studies consisted of only one phospholipid type and, in general, had a very simplified structure. Therefore, conclusions based on the results have some limitations. One must realize that the lysophospholipid interaction with cell membranes depends on their components, complex structure, and physiologic state. Our previous studies confirmed different ways of LPC incorporation into the lipid bilayer of βTC3 cells and even cytotoxic effects caused by LPC 16:0, while LPC 12:0 and LPC 14:0 with shorter acyl chains had no toxic effects [76]. LPC concentration used in our studies with βTC3, MIN6, and psudoislets were lower than those showing cytotoxic effects. Therefore, we did not observe any toxic effect, but the only different level of GSIS in the presence of these LPCs, probably caused by a different way of their binding to membranes and the receptors.

In summary, we have compared some aspects of GPCR-related functional properties of βTC3 and MIN6 cell lines, grown either as adherent monolayers or forming pseudoislets with pancreatic MS1 endothelial cells. We show that the co-culture of MS1 with the chosen pancreatic β cell models brings about uniformization of *Gpr40, Gpr55,* and *Gpr119* expression patterns and changes the sensitivity to treatment with their LPC ligands. It proves the imperfection of traditional monolayered β cell models and presents the possible GPCR-related mechanisms responsible for the impaired response. However, further studies taking into account other GPCRs, e.g., GLP1R, are necessary to demonstrate the improved functionality of PIs. Moreover, the observed varying efficiencies in GSIS potentiation between LPCs bearing various acyl chains could suggest their multiple applications.

## Figures and Tables

**Table 1:** Primers used in the study.

Gene Symbol	Forward Primer	Reverse Primer	Accession No.
*Ins1*	5′-ACCTGGAGACCTTAATGGGCCAAA-3′	5′-ATGACCTGCTTGCTGATGGTCTCT-3′	NM_008386.3
*Actβ*	5′-AAGAGCTATGAGCTGCCTGA-3	5′-TACGGATGTCAACGTCACAC-3′	NM_007393.5
*Gpr40*	5′-TCTGCCTGGGGCCCTATAAT-3′	5′-TCCAGGACCTGTTCCCAAGT-3′	NM_194057.3
*Gpr55*	5′-AGCCTTCTGACTTGGACAGC-3′	5′-CCTCATCCCCTTCATACTGG-3′	NM_001033290.2
*Gpr119*	5′-CTTCTACTGTGACATGCTCAAGATTG-3	5′-CCATGGCTCCTGCATGTTC-3	NM_181751.2
